# The anti-inflammatory effects of curcumin on renal ischemia-reperfusion injury in rats

**DOI:** 10.1080/0886022X.2018.1544565

**Published:** 2018-12-04

**Authors:** Jiong Zhang, Li Tang, Gui Sen Li, Jia Wang

**Affiliations:** a Department of Nephrology, University of Electronic Science and Technology, Sichuan Academy of Sciences & Sichuan Provincial People's Hospital, Chengdu, China;; b Department of Nephrology, Zigong Third People's Hospital, Zigong, China;; c General Medicine Center and University of Electronic Science and Technology, Sichuan Academy of Sciences & Sichuan Provincial People's Hospital, Chengdu, China

**Keywords:** Curcumin, renal ischemia reperfusion injury, inflammation

## Abstract

The present study aimed to explore the protective mechanism of curcumin-precondition in renal ischemia-reperfusion (I/R) injury. Thirty male SD rats were randomly equally divided into Sham, IRI, and Curcumin groups (*n* = 10). The model of IRI was induced by clamping the left renal artery for 45 min followed by 24 h reperfusion with the contralateral nephrectomy. ELISA was used to examine the expression of Cr, BUN, IL-8, TNF-α, and IL-6 in the serum; RT-PCR was used to detect the mRNA level of IL-8,TNF-α, and IL-6 in the kidney; The morphology of kidney was examined by Periodic Acid-Schiff stain (PAS). The expression of JAK2, p-JAK2, STAT3, p-STAT3, p65, and p-p65 in kidney were detected by Western blotting. The result displayed that Curcumin pretreatment can significantly increase the expression of p-JAK2 and p-STAT3 and reduce the expression of Cr, BUN, IL-8, TNF-α, IL-6, and p-p65. In addition, Curcumin can attenuate the kidney pathological injury and have no effect on the expression of JAK2, STAT3, and p65. Our findings showed the protective effect of Curcumin in I/R injury is associated with suppressing NF-κB mediating inflammation by activating JAK2/STAT3 signal pathway.

## Introduction

Renal ischemia is commonly seen in patients with cardiovascular surgery, trauma, shock, burn, and organ transplantation [1,2]. The ischemia-reperfusion (I/R) injury causes molecular and cellular inflammatory response within the kidney, such as activation of inflammatory-relevant transcription factor, nuclear factor-κB (NF-κB) which plays an important role in pathogenesis of I/R injury [3]. Important to note that increased activation of NF-κB in the I/R-challenged kidney further contributes to kidney tissue damage often causing systemic inflammatory response and subsequent leading to acute kidney failure [4].

Curcumin is the major active component of *Curcuma longa* and wildly used as spice and edible pigment in daily life [5], which has been shown to act pharmacologically in cerebral tissue where Curcumin exerted anti-inflammatory effects [5,6]. However, the role and potential molecular mechanisms of Curcumin in modulation of I/R-induced inflammatory response in the kidney has been uncertain, yet. Therefore, in this study, we explore the effects and potential mechanisms of Curcumin in modulation of I/R-induced inflammatory response in the kidney of rats.

## Methods

### Experimental model

All experiments in this study were authorized by the Institutional Animal Care and Use Committee at the University of Electronic Science and Technology, Sichuan Academy of Sciences, and Sichuan Provincial People's. Thirty male Sprague-Dawley rats were kept in a standard SPF condition with a 12 h dark/light cycle, and were allowed to eat food and drink water freely. Animas were anesthetized by intraperitoneal injection of pentobarbital sodium (50 mg/kg) and kept core body temperature at 37 °C by a recirculating water bath mat. Moreover, a midline laparotomy was performed to fully explore bilateral renal arteries and veins. The left renal pedicle was occluded with a non-invasive microvascular clamp for 45 min. As soon as the ischemic time arrives, the clamp are loosened immediately for 24 h blood reperfusion with contralateral nephrectomy. After 24 h reperfusion, the rats were put to death to collect serum and renal tissue.

### Experimental groups

The rats were randomly equally divided into three groups (*n* = 10 for each group):

1. I/R-Curcumin group (Cur) in which the rats were subjected to renal ischemia for 45 min and precondition with 60 mg/kg (i.p. injection) of Curcumin (Sigma, St. Louis, MO) at 45 min before I/R induction; The dosage of Curcumin was in line with previous study [7].
2. I/R-vehicle group (IRI), in which the rats were subjected to renal ischemia for 45 min and precondition with 60 mg/kg (i.p. injection) of saline (Sigma, St. Louis, MO) at 45 min before I/R induction.
3. Sham-operated group (Sham), in which the rats were subjected to identical surgical procedure as above but without renal I/R injury.

### Evaluation of renal function

The whole blood of rats were centrifuged for 10 min at 2–8 °C for 3000 rpm/min, then the upper serum was collected and stored at –80 °C for reserve. The serum of rats was taken and sent to the Laboratory of Sichuan Provincial People's Hospital to detect the levels of creatinine (Cr) and urea nitrogen (BUN) in the serum for assessing renal function.

### Determination of histological morphology

The renal sample were fixed in 4% formalin for 12 h followed by paraffin embedding, then the renal tissues were cut into 4–5 μm sections. The sections were subjected to PAS staining and were scored by a pathologist using a double-blind method under a microscope. The specific scoring standard was in base on reference [8].

### Western blot of JAK2, p-JAK2, STAT3, p-STAT3, p65, and p-p65

The renal tissue was homogenized followed by collecting supernatants to examine the protein concentrations by the method of BCA. Proteins were separated by SDS-PAGE and transferred to polyvinylidene fluoride membranes. The membranes were then incubated with antibodies against p-p65 (Abcam, Cambridge, UK, 1:500), p65 (Abcam, Cambridge, UK, 1:1000), p-JAK2 (CST, Boston, MA, 1:500), JAK2 (CST, Boston, MA, 1:1000), p-STAT3 (CST, Boston, MA, 1:500), STAT3 (CST, Boston, MA, 1:500), and β-actin (Abmart, Shanghai, China, 1:3000) one night at °C, followed by incubation with an HRP-conjugated secondary antibody (Abmart, Shanghai, China, 1:3000) at room temperature for 2 h . The reactive bands were visualized by use of the ECL-Plus reagent (Amersham, Piscataway, NJ). The density of band was quantified by the Quantity One analytic software (Upland, CA).

### Determination of the mRNA level of TNF-α, IL-6, and IL-8

Total RNA was isolated from the whole renal tissues by use of Trizol (Takara, Japan) following the manufacturer’s instructions. Total RNA were then reverse by using a commercial kit (Takara, Japan). Real-time PCR amplifications were implemented by using the ABI 7500 system (Thermo ElectronCorporation, MA, USA). The primers (Invitrogen, Carlsbad, CA) for RT- PCR are displayed in Table 1. The reverse transcription for RT-PCR was carried out at 95 °C for 30 s, following 40 cycles at 95 °C for 5 s, then 60 °C for 34 s, and 95 °C for 15 s. The content of mRNA was normalized by β-actin and the relative expression were calculated by the 2^−ΔΔCt^ method as described in instructions.

**Table 1.** The primers used for real-time PCR analysis.

Gene	Species	Sense strand sequence	Anti-sense strand sequence
TNF-α	rat	AACACGAGTGACAAGCCCGTAG	GTAT CACCAGTTGGTTCTCTTTGA
Il-6	rat	AGGTTCCATGTGCAAGTGTCT	GACAGCCCTGGTCAAAGGTT
IL-8	rat	CTGCAAGAGACTTCCATCCAG	AGTGGTATAGACAGGTCTGTTGG
β-actin	rat	AGAGGGAAATCGTGCGTGAC	CAATAGTGATGACCTGGCCGT

### Determination of the level of TNF-α, IL-6, and IL-8 in the serum

The concentration of TNF-α, IL-6, and IL-8 in the serum was detected by a commercially available ELISA kit for rats following the manufacturer’s instructions (Biosource International Inc., Camarillo, CA).

### Statistical analysis

Values are expressed as mean ± SEM. Group comparisons were processed by using the Student’s *t*-test or the one-way analysis of variance (ANOVA) test. In all cases, *p* < 0.05 was considered as statistically significant.

## Results

### Curcumin precondition improved renal function

**Figure 1.**
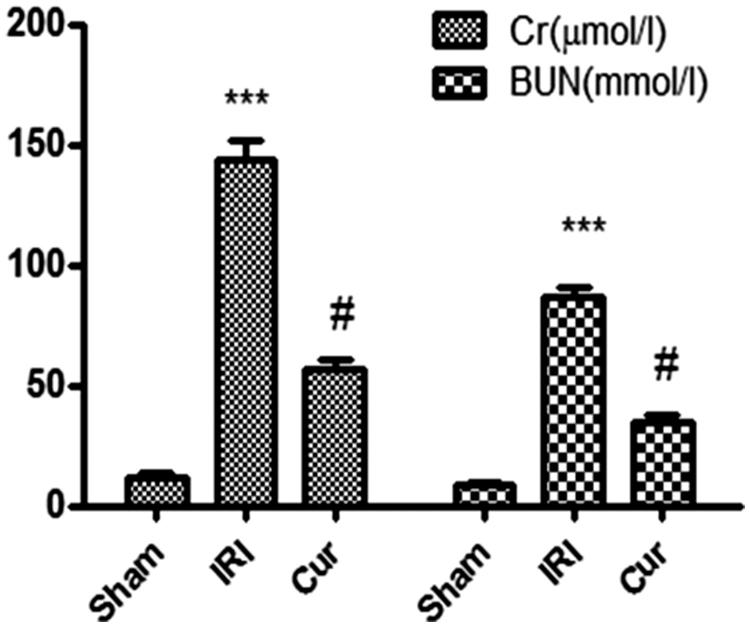
Effects of Curcumin pretreatment on renal function following renal I/R-induced injury. Serum creatinine and BUN were measured to assess the reno-protective effect of Cur against renal I/R. Data are represented as mean ± SEM (*n* = 10). ***p < .001 (IRI vs. Sham); ^#^*p* < .005 (IRI vs. Cur).

In IRI group, the content of Cr and BUN in serum were significantly higher than those in the Sham group (*p* < .001), while levels of Cr and BUN were obliviously lower in Cur group than those in IRI group (*p* < .001) (Figure 1).

### Curcumin precondition alleviated kidney pathological injury

**Figure 2.**
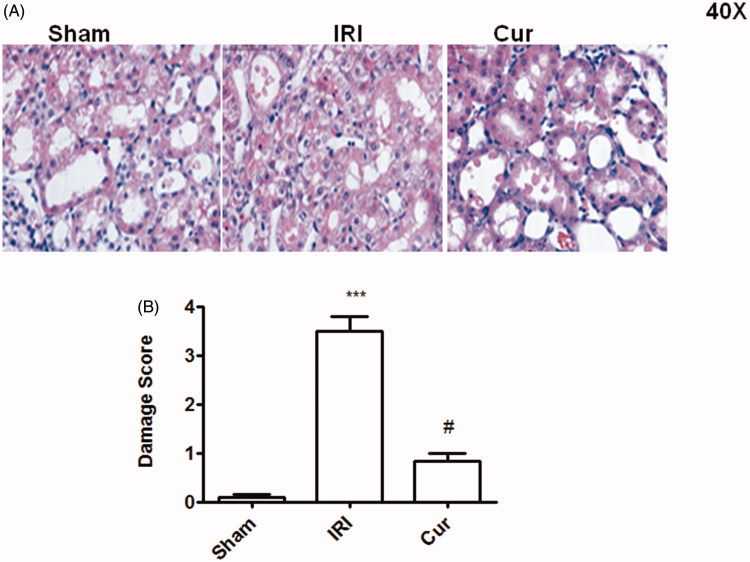
Effects of Curcumin pretreatment on I/R-induced renal histology. Representative microphotographs were taken from the kidneys of the sham, IRI, and Cur groups at the time point of 24 h after renal I/R. Histopathological examination was performed using PAS staining. Semi-quantitative assessment of the histological lesions based on tubular necrosis (B). Data are represented as mean ± SEM (*n* = 10). ***p < .001 (IRI vs. Sham); ^#^*p* < .001 (IRI vs. Cur).

The Sham group showed almost normol pathological structure with no injury in kidney (Figure 2(A)). The IRI group displayed serious pathological change with water-like or vacuolar degeneration of renal tubular epithelial cells, disappearance of brush-like margin, coagulative necrosis, and exfoliation of some renal tubular epithelial cells, tubular type, interstitial edema and focal inflammation in the interstitium (Figure 2(B)). However, the renal pathological changes in Cur group were significantly alleviated compared with those in IRI group (Figure 2(B)). The histopathological score of kidney in three groups is as shown in Figure 2(D).

### Curcumin pretreatment reduced the mRNA level of TNF-α, IL-8, and IL-6

As shown in Table 2, the mRNA level of TNF-α, IL-8, and IL-6 in IRI group was significantly higher than those in Sham group (*p* < .05). However, precondition with Cur can obliviously decrease the mRNA level of TNF-α, IL-8, and IL-6 as displayed lower mRNA level of TNF-α, IL-8, and IL-6 in Cur group than IRI group (*p* < .05).

**Table 2.** Effects of curcumin pretreatment on the mRNA level of TNF-α, IL-6, and IL-8 in the kidney after renal ischemia-reperfusion injury.

Group	Number	TNF-α (mRNA)	IL-6 (mRNA)	IL-8 (mRNA)
Sham	10	1 ± 0.1	1 ± 0.05	0.95 ± 0.1
IRI	10	12 ± 1.5***	5 ± 0.4***	7.5 ± 0.75***
Cur	10	5 ± 0.5^#^	1.5 ± 0.5^##^	2.5 ± 0.4^#^

Data are represented as mean ± SEM (*n* = 10).

***p < .001 (IRI vs. Sham); ^##^*p* < .001, ^#^*p* < .05 (IRI vs. Cur).

**Table 3.** Effects of curcumin pretreatment on the expression of TNF-α, IL-6, and IL-8 in the serum after renal ischemia-reperfusion injury.

Group	Number	TNF-α (pg/ml)	IL-6 (pg/ml)	IL-8 (pg/ml)
Sham	10	100 ± 10	120 ± 20	100 ± 20
IRI	10	1200 ± 200***	550 ± 50***	750 ± 50***
Cur	10	400 ± 150^##^	200 ± 30^#^	350 ± 40^#^

Data are represented as mean ± SEM (*n* = 10).

***p < .001 (IRI vs. Sham); ^##^*p* < .01, *p* < .05 (IRI vs. Cur).

### Curcumin precondition decreased the secretion of TNF-α, IL-8, and IL-6

In IRI group, the content of TNF-α, IL-8, and IL-6 in serum were significantly higher than those in Sham group (*p* < .05), while levels of TNF-α, IL-8, and IL-6 in serum were obliviously lower in Cur group than those in IRI group (*p* < .05), which indicated that Curcumin precondition decreased the secretion of TNF-α, IL-8, and IL-6 in IRI (Table 3).

### Curcumin pretreatment suppressed NF-κB activation

**Figure 3.**
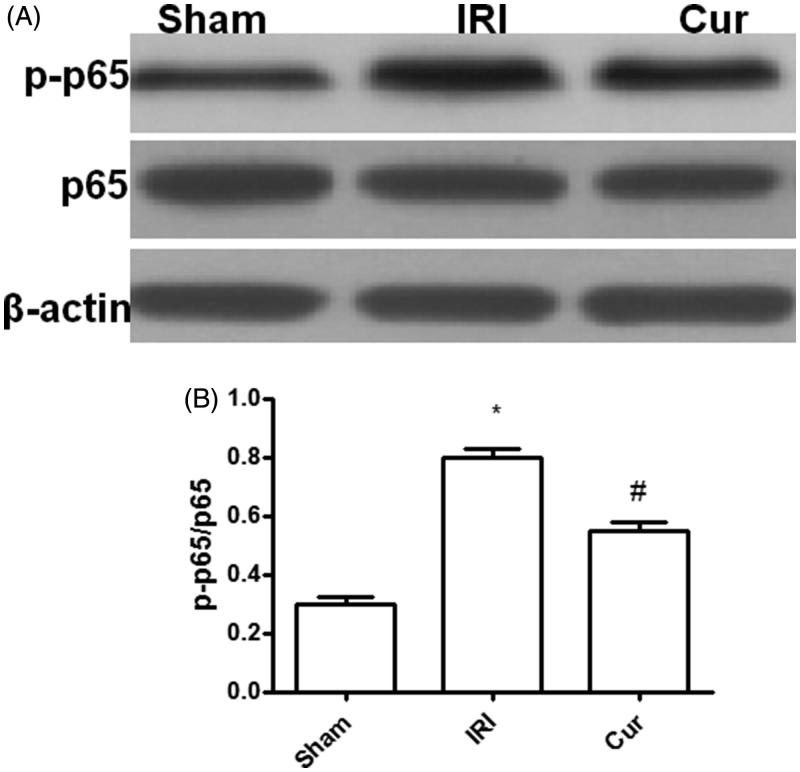
Effects of Curcumin pretreatment on the expression of p65 and p-p65 after renal ischemia-reperfusion injury. Western blot analysis was employed to the expression of p65 and p-p65. (A) A representative result for Western blot analysis p65. (B) Semi-quantitative analysis of 10 animals studied in each group. The relative amounts of p-p65 and p65 in each group of rats were normalized by β-actin and presented as a ratio between p-p65 and p65. *p < .05 (IRI vs. Sham); ^#^*p* < .05 (Cur vs. IRI).

NF-κB plays an important role in inflammatory response in IRI and its activation is relayed on p65 activation [3,4]. As shown in the figure neither IR insult nor Curcumin pretreatment showed impact on p65 expression (Figure 3(A,B)). However, the amount of activated p65 (p-p65) noted in rats after IR insult was significantly higher as compared with that in Sham rats (Figure 3(A,B)). Nevertheless, pretreatment with Curcumin can significantly decrease p65 activation as showed at lower levels of p-p65 in Cur group as compared with that in IRI group. In all, our result suggest that Curcumin precondition can suppress inflammatory response in IRI by attenuating NF-κB activation (Figure 3(A,B)).

### Curcumin precondition promoted the activation of JAK2/STAT3 signal

**Figure 4.**
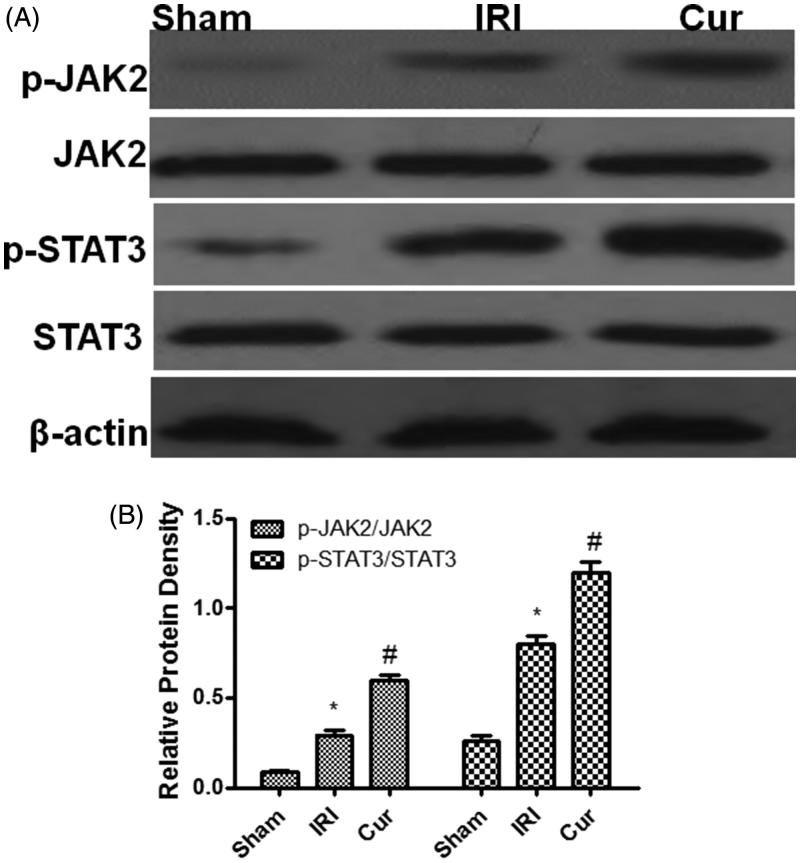
Effects of Curcumin pretreatment on the JAK2/STAT3 signaling after renal ischemia-reperfusion injury. Western blot analysis was employed to the expression of JAK2, p-JAK2, STAT3, and p-STATA3. (A) A representative result for Western blot analysis p-JAK2, STAT3, and p-STATA3. (B) Semi-quantitative analysis of 10 animals studied in each group. The relative amounts of p-JAK2, JAK2, STAT3, and p-STATA3 in each group of rats were normalized by β-actin and presented as a ratio between p-JAK2/JAK2 and p-STAT3/STAT3. *p<.05 (IRI vs. Sham); ^#^*p*<.05 (Curcumin vs. IRI). (IRI vs. Sham); ^#^*p*<.05 (Cur vs. IRI).

In order to explore the mechanism of Curcumin suppressing NF-κB activation in IRI, we selectively analyzed the signal of JAK2/STAT3 pathway. As shown in Figure 4, the result displayed compared with the Sham group, the expression of p-JAK2 and p-STAT3 are higher than those in Sham group, which implied IRI can induce the activation of JAK2/STAT3 signal (*p* < .05); It is noteworthy that Cur precondition can further promote the activation of JAK2/STAT3 signal as showed at higher level of p-JAK2 and p-STAT3 in Cur group than those in the IRI group. Moreover, there is no difference in the JAK2 and STAT3 expression among three groups. Collectively, our study indicated that Curcumin precondition can advance the signal pathway JAK2/STAT3 activation in IRI.

**Figure 5.**
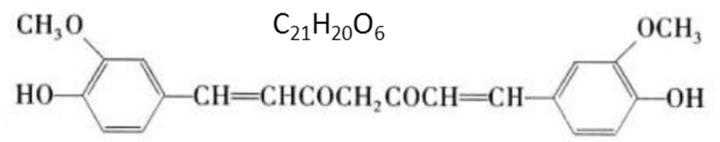
The structure of curcumin.

## Discussion

Renal I/R injury is one of the main pathogenesis of acute renal injury and inflammation is an important pathophysiological mechanism of IRI. IRI can induce inflammatory cell aggregation, release of inflammatory factors (such as TNF-α, IL-8, and IL-6), and increase of adhesion molecules. These factors together stimulate the cascade reaction of inflammation, leading to inflammation and organ damage [3,4].

Our study showed that the sham operation had no influence on common renal parameters such as renal function, pathomorphology, and inflammation. IRI can lead to severe renal impairment and renal pathological changes in rats. In accordance with the above change, IRI also significantly increase the expression of inflammatory factors TNF-α, IL-8, and IL-6 [9], which further demonstrated that the inflammation play a vital role in IRI and suppressing inflammation is an effective target to decrease renal IRI.

Curcumin has been shown that can reduce cerebral ischemia reperfusion injury by anti-inflammation [6] and its structure is as shown in Figure 5. However, to date, there is still a lack of precise study on the protective mechanisms of Curcumin in renal I/R injury. In the present study, we further demonstrated that Curcumin can protect rats against renal ischemia reperfusion injury as manifested attenuating renal dysfunction and the histopathology alteration. The mechanism of which is associated with suppressing inflammation as displayed decreasing the expression of pro-inflammatory cytokine TNF-α, IL-8, and IL-6 induced by renal IR injury.

NF-κB is a nuclear transcription factor that exists widely in cytoplasm [9]. It is composed of two subunits, P50 and p65 and its main functional unit is p65. It has the ability to promote inflammation [9]. Under the resting state, the complex of NF-κB and IκB-α were distributed in cytoplasm in an inactive form. If stimulated by signaling molecules, IκB-α can be phosphorylated, and then the complex can be dissociated, causing NF-κB to migrate to the nucleus, combining with targeted DNA sequences, promoting the expression of downstream pro-inflammatory genes, and the release of pro-inflammatory factors such as TNF-α, IL-8, and IL-6, which can further promote the activation of NF-κB [9,10]. The regulation of positive feedback results in a cascade of inflammatory reactions [9,10]. In our study, we found both IRI and Cur have no impact on the p65 expression. But IRI can induce the activation of p65 as displayed higher expression of p-p65 in IRI group than that in Sham group. Moreover, Cur precondition can suppress the activation of p65 as displayed less expression p-p65 in Cur group than that in IRI group which suggested that Curcumin precondition can significantly alleviate NF-κB activation in IRI.

Studies have shown that one IRI can initiate a variety of signaling pathways, among which highly conserved JAK2/STAT3 signaling pathways can be reactivated and participate in the regeneration and repair of renal tubular epithelial cells by suppressing NF-κB activation [11,12]. Therefore, we examine the effect of Curcumin on JAK2/STAT3 signaling in IRI. The result displayed that neither Cur nor IRI has impact on the JAK2 and STAT3 expression, but IRI can induce the activation of JAK2/STAT3 signaling as displayed higher expression of p-JAK2 and p-STAT3 in IRI group than those in Sham group. Surprising, Cur precondition can further promote the activation of JAK2/STAT3 sigaling as displayed more expression p-JAK2 and p-STAT3 in Cur group than that in IRI group which suggested that Curcumin precondition can promote the activation of JA2/STAT3 signaling by phosphorylation modification in IRI. Because there is a research implying that activating JAK2/STAT3 signal pathway can attenuate IRI [13], therefore, in the study we did not carried out additional studies to demonstrate that inhibiting JAK2/STAT3 signal pathway can promote NF-κB activation in IRI. Given the capacity of Cur pretreatment in attenuating IRI, it is noteworthy that Curcumin could mediate another pathway to inhibit NF-κB activation besides JAK2/STAT3 signal pathway, such as the PI3K/AKT cascade [14]. Therefore, further studies with focus addressing the pathways associated with inhibiting NF-κB activation by Curcumin in IRI would be necessary.

In summary, we demonstrated under convincing evidence that the protective effect of Curcumin in renal I/R injury is associated with suppressing NF-ΚB mediating inflammation by activating JAK2/STAT3 signal pathway. Altogether, our data support that Curcumin could be a good alternative therapy for preventing renal ischemia reperfusion injury in the clinical practice.
